# Association between obesity and periodontal disease in young adults: a population-based birth cohort

**DOI:** 10.1111/j.1600-051X.2012.01906.x

**Published:** 2012-06-03

**Authors:** Eduardo Dickie de Castilhos, Bernardo Lessa Horta, Denise Petrucci Gigante, Flávio Fernando Demarco, Karen Glazer Peres, Marco Aurélio Peres

**Affiliations:** 1 Post-Graduate Program in Epidemiology, Pelotas Federal UniversityPelotas, Brazil; 2 Department of Public Health, Post-Graduate Program in Public Health, Center for Health Sciences, Federal University of Santa CatarinaFlorianópolis, Brazil

**Keywords:** body mass index, cohort studies, C-reactive protein, periodontal diseases, waist circumference

## Abstract

**Aim:**

To evaluate the association between obesity and periodontal disease and the mediating effect of oral hygiene, systemic inflammation and carbohydrate intake.

**Material and methods:**

Subjects born in 1982 in Pelotas, Brazil (*n* = 5,914), have been followed for several times. Oral health was assessed in a representative sample of 720 individuals at 24 years. Obesity, waist circumference and number of episodes with obesity between 15 and 23 years of age were the main exposures. Mediating effect of oral hygiene, C-reactive protein level and carbohydrate consumption was also assessed.

**Results:**

Obese individuals were more likely to have ≥2 teeth with gingival bleeding. However, after adjusting for confounders, the association was not statistically significant [OR (obese × 2 or more teeth) 1.72 (95% CI: 0.95, 3.11)] and adjustment for potential mediators decreased the OR (OR = 1.38). The risk of presenting calculus in obese subjects was 10% higher [PR 1.10 (95% CI: 1.02, 1.18)]. The number of episodes of obesity between 15 and 23 years was associated with dental calculus. Periodontal pockets were not associated with obesity.

**Conclusion:**

Systemic inflammation and oral hygiene may be mediating the association between obesity and gingivitis. Obesity was not associated with periodontal pockets in young adults in this cohort.

**Conflict of interest and source of funding statement**

The authors declare that there are no conflicts of interest.

This article is based on data from the study “Pelotas birth cohort, 1982” conducted by Postgraduate Program in Epidemiology at Universidade Federal de Pelotas. The 1982 birth cohort study is currently supported by the Wellcome Trust Initiative entitled Major Awards for Latin America on Health Consequences of Population Change. Previous phases of the study were supported by the International Development Research Center, The World Health Organization, Overseas Development Administration, European Union, National Support Program for Centers of Excellence (PRONEX), the Brazilian National Research Council (CNPq) and Brazilian Ministry of Health. The Oral Health Studies were supported by the Brazilian National Research Council (CNPq) – Research Financing (Process 479621/20047 – Flávio Fernando Demarco and Process 476985/20045 – Karen Glazer Peres).

It has been suggested that obesity is related to the occurrence of periodontal disease. Cross-sectional studies have reported the association between obesity and periodontal diseases (Wood et al. 2003), as well as with periodontitis (Al-Zahrani et al. 2003, Dalla Vecchia et al. 2005, Genco et al. 2005, Nishida et al. 2005, Saito et al. 2005, Ekuni et al. 2008, Khader et al. 2009, Kongstad et al. 2009) and gingivitis. It has also been reported that obesity is related to the presence of risk factors for periodontal disease, such as biofilm and dental calculus (Borges-Yanez et al. 2006, Franchini et al. 2011). On the other hand, findings from cohort studies are heterogeneous. Morita et al. (2011) reported that periodontal disease was positively associated with body mass index (BMI) 5 years earlier, whereas Linden et al. (2007) did not observe an association between obesity in early adulthood and severity of periodontitis at the age of 60–70 years. A systematic review of Suvan et al. (2011) observed a heterogeneity among studies, but all of them have reported a higher odds of periodontitis among obese individuals [pooled odds ratio 2.13 (95% CI: 1.40; 3.26). Most of the studies were carried among adults and elderly people in the literature search, and we did not identify any study that have assessed whether obesity cause periodontal disease among young adults. It is important to evaluate this association among young adults to better understand the early systemic consequences of obesity.

Obesity is associated with a systemic inflammatory state, and elevated levels of C-reactive protein, a marker of low-grade inflammation, have been reported in obese individuals (Visser et al. 1999, Rexrode et al. 2003, Nazmi et al. 2008). This low-grade inflammation would be one of the mechanisms through which obesity triggers the installation and/or worsening of non-transmissible chronic diseases (Brooks et al. 2010), and it would also be related to the occurrence of periodontal disease (Pischon et al. 2007). Pischon et al. (2007) suggested that the secretion of inflammatory cytokines by adipose tissue could be triggered by lipopolysaccharide of gram-negative periodontal bacteria, leading to hepatic dyslipidemia and reduction in insulin sensitivity. This reaction would be enhanced in individuals with higher amounts of adipose tissue and would exacerbate the systemic inflammatory condition predisposing to the establishment or aggravation of inflammatory diseases such as periodontitis. The knowledge about how molecular and cellular pathways are related to periodontal disease is far from complete, requiring other studies (Preshaw & Taylor 2011). On the other hand, obesity is also associated with risk factors for periodontal disease, for example, consumption of carbohydrates is higher among obese subjects (Hujoel 2009). Therefore, obesity could be a marker of a risky behaviour for periodontal disease, not a risk factor (Genco et al. 2005, Hujoel 2009).

As previously mentioned, many studies have observed an association between obesity and periodontal disease, but the possible mechanisms for such association have poorly assessed. Saito et al. (2005) observed that the association between obesity and periodontal pocket was observed even after controlling for glucose tolerance. Therefore, insulin resistance would not be a mediator in the association between obesity and periodontal disease.

Concerning the assessment of causality, there are few cohort studies addressing this issue. Cohort studies have the advantage of clearly defining the temporality of the association and are less susceptible to measurement error and residual confounding. Therefore, cohort studies provide more strong evidence on the existence of an association. This study aimed to assess the association between obesity among young adults and periodontal disease and the mediating effect of oral hygiene, systemic inflammation and carbohydrate intake.

## Methods

In 1982, the three maternity hospitals in Pelotas, a southern Brazilian city, were visited daily and 7392 births were identified. Those children whose parents lived in the urban area of the city (*N* = 5914) were examined and the mothers interviewed. These individuals have been followed on several occasions (Barros et al. 2008).

In 1997, a census was carried out in a systematic sample of 70 from the 259 census tracts located in the urban area of the city, and subjects born in 1982 were linked to the study records and those subjects belonging to the cohort were interviewed and examined (*n* = 1,076). In 2004–2005, we tried to follow up the whole cohort and the subjects answered a questionnaire on sociodemographic, health and behavioural variables. At the end of the interview, the subjects were invited to visit the research laboratory to give a blood sample. Concerning the assessment of oral health, a sample of 900 individuals examined in 1997 was randomly selected to participate in the oral health study (OHS-97). Of the 900 selected subjects, 888 (98.7%) agreed to participate in the OHS-97 (Peres et al. 2011). In 2006, those individuals who participated in the OHS-97 were again contacted, 720 of 888 individuals (81.1%) were located and participated in the OHS-06. In the OHS-06, all teeth were examined for the presence of periodontal diseases (gum bleeding, dental calculus and periodontal pocket), dental caries (DMFT), quality of restorative treatments, oral cavity lesions and use/needs of dental prosthesis. The interview covered issues related to hygiene, use of dental services and dental pain. In 2006, oral examination was performed at home by six dentists and four students at final year of graduation in dentistry, previously trained and calibrated. Examiners underwent theoretical and practical training covering diagnostic criteria and details on each index. Practical exercises were performed on 20 patients, and reproducibility assessment was performed on 25 volunteers. Diagnostic inter-examiner reproducibility was measured, and the lowest Kappa was 0.60 for gingival bleeding but most of the values were close to 1.00. More details were reported elsewhere (Peres et al. 2011).

The phases of the study were approved by the Ethical Review Board of the Faculty of Medicine of the Federal University of Pelotas, and written informed consent was obtained from participating subjects.

### Definition of outcomes

All teeth were examined at six different sites (mesiobuccal, mediobuccal, distobuccal, mesiolingual, mediolingual and distolingual). The following periodontal disease outcomes were assessed:

- Gingivitis: all sites were probed, waiting 10 s to verify the presence or absence of gingival bleeding. The variable was categorized as absent, a tooth and two or more teeth with gingival bleeding.
- Calculus: all sites were probed for detection of calculus. The variable was dichotomized in absence or presence of dental calculus. Presence was considered if calculus was present in at least one surface.
- Periodontal pocket: all sites were probed, pocket should have probing depth ≥4 mm in at least one site. The variable was dichotomized in absence or presence of periodontal pocket. Presence was considered if periodontal pockets were present in at least one surface.

For each outcome, the results were recorded in separate forms for each tooth in relation to the outcomes.

### Definition of exposures

In each visit, the subjects were weighted and their height was assessed. BMI was estimated in kg/m^2^. Waist circumference was measured in 2004–2005 and measured at the narrowest part of the trunk directly on the skin. For individuals with no visible waist circumference, this measure was made at the midpoint between the iliac crest and last rib. Measures of waist circumference were categorized according to sex in normal (men < 94 cm, women < 80 cm), level 1 (men ≥ 94 and <102 cm, women ≥80 and <88 cm) and level 2 (men ≥ 102 cm; women ≥ 88 cm; Lean et al. 1995).

At the age of 15 years, the following cut-off was used to categorize the BMI: eutrophic (BMI in *z* score for age and sex ≤1 SD), overweight (BMI > 1 and <2 SD) or obesity (BMI ≥ 2 SD; World Health Organization 2007). At 18 and 23 years, the following cut-off were used to categorize BMI eutrophic (BMI < 25 kg/m^2^), overweight (BMI ≥ 25 and ≤29.9 kg/m^2^) and obesity (BMI ≥ 30 kg/m^2^; World Health Organization 1998).

In the follow-up visits at 15, 18 and 23 years, individuals were classified as obese or non-obese according to WHO criteria previously cited. We created a variable that summarized the number of times that each subject was considered obese. Therefore, the individuals could have been classified as obese in none, one follow-up and two or more follow-ups with obesity.

### Confounders and mediators variables

The following variables were considered possible confounding factors:

Sex, skin colour [classified as white and black (black + brown)], smoking at 23 years (never smoked, former smoker and smoker), attained schooling at 23 years (classified according to the highest level of schooling in years at 0–4, 5–8, 9–11, 12 or more), family income at the age of 23 years (categorized into tertiles according to the total family income in the month preceding the interview), and the asset index at 23 years of age was estimated by factor analysis from scores of household goods such as vacuum cleaner, washing machine, DVD, fridge, freezer, microwave oven, computer, telephone, radio, television, automobile and air conditioning. This variable also included salaried housemaid, maternal education and family income. From the different components generated in the factor analysis, the first was used to create a continuous score that included all variables who contributed directly. This variable was subsequently divided into tertiles.

The following possible mediating factors were also included in the analysis: use of dental floss (yes/no); reported frequency of brushing (0–2, 3 and 4 or more times a day) at age 24 years; percentage of dietary energy intake from carbohydrates at age 23 years categorized in tertiles; and C-reactive protein level at age 23 years (low ≤ 1.0 mg/l, moderate from 1.01 to 3.0 mg/l and high 3.01–10.0 mg/l). C-reactive protein values greater than 10.0 mg/l were excluded from the analysis as they are related to acute inflammation, not chronic (Pearson et al. 2003). In the 2004–2005 visit, the subjects answered a food frequency questionnaire, and the percentage of dietary energy from carbohydrates was estimated (Olinto et al. 2011).

### Statistical analysis

Descriptive analysis of the population was performed using absolute and relative frequencies and Fisher's exact test. Multinomial logistic regression was used to assess the relationship between number of teeth with gingivitis (none, 1 or ≥2). Poisson regression was used to estimate the prevalence ratio of calculus and periodontal pocket (Barros & Hirakata 2003).

All potential confounding factors which showed *p* < 0.2 were selected to remain in the multivariable analysis.

Effect modification by gender, income and smoking was evaluated with interaction terms in the multivariate analysis. Analyses were performed with software STATA 11.0 for Windows, StataCorp., College Station, Texas.

## Results

In 2006, 720 subjects were examined (participation rate 81.2%); the prevalence of gingivitis, calculus and periodontal pocket was 37.5%, 87.4% and 3.3%, respectively. Table 1 describes the sample according to socioeconomic, demographic characteristics, oral hygiene habits and health-related behaviours. Most individuals were white (71.3%) and had achieved at least 8 years of schooling. With regard to health behaviours, 23.0% were smokers at age 23. Regarding to oral hygiene habits, about half of the subjects reported the use of dental floss, and 98.2% used to brush the teeth at least three times a day.

**Table 1:** Distribution of studied sample according to demographic, socioeconomic, health behaviour and oral hygiene variable at 23 and 24 years of age. Pelotas, RS

	Prevalence			*n*
		
	Gingival bleeding (%)	Dental calculus (%)	Periodontal pocket (%)	
Gender	*p* = 0.817	*p* = 0.217	*p* = 0.675	
Male	38.2	88.9	2.9	379
Female	37.1	85.8	3.6	339
Skin colour	*p* = 0.105	*p* = 0.170	*p* = 0.818	
White	35.8	86.2	3.2	511
Black	42.4	90.2	3.4	206
Achieved schooling at 23 years of age	*p* < 0.001	*p* = 0.033	*p* = 0.420	
0–4 years	61.0	90.2	4.9	42
5–8 years	43.8	92.4	4.9	186
9–11 years	33.5	84.6	2.8	365
12 or more	29.0	86.0	2.0	101
Family income at 23 years of age	*p* = 0.049	*p* = 0.446	*p* = 0.023	
1st tertile	43.2	89.8	6.3	208
2nd tertile	37.2	86.6	1.9	269
3rd tertile	31.6	86.1	2.3	217
Assets index at age 23 in tertiles	*p* = 0.238	*p* = 0.034	*p* = 0.166	
First (lower)	41.0	92.2	5.1	219
Second	35.4	84.8	3.0	237
Third (higher)	33.5	86.0	1.8	222
Smoking at 23 years	*p* = 0.975	*p* = 0.015	*p* = 0.648	
Never smoked	37.0	85.2	3.2	478
Ex-smoker	37.9	87.9	5.2	56
Smoker	37.7	93.7	3.1	160
Body mass index at 23 years of age	*p* = 0.219	*p* = 0.095	*p* = 1.000	
< 25 kg/m^2^	35.2	85.7	3.4	481
≥ 25 and ≤29.9 kg/m^2^	40.5	89.5	3.3	153
BMI ≥ 30 kg/m^2^	44.8	94.8	3.5	58
Waist circumference at 23 years of age	*p* = 0.131	*p* = 0.185	*p* = 0.424	
Normal	35.5	86.6	3.1	562
Level 1	46.5	87.3	5.6	71
Level 2	42.4	94.9	3.4	59
Dental flossing report at 24 years of age	*p* < 0.001	*p* = 0.018	*p* = 0.398	
No	45.9	90.3	3.9	364
Yes	29.3	84.4	2.6	354
Daily brushing report at 24 years of age	*p* = 0.046	*p* = 0.054	*p* = 0.562	
≤2	66.7	91.7	-	13
3	41.3	92.1	2.1	190
≥4	35.7	85.6	3.7	515
C-reactive protein level	*p* = 0.171	*p* = 0.123	*p* = 0.483	
≤1.0 mg/l	35.4	85.8	2.8	289
≥1.01 and ≤ 3.0 mg/l	36.3	85.4	2.9	171
≥3.01 and ≤ 10.0 mg/l	44.6	92.1	5.0	141
Percentage of calories from carbohydrates	*p* = 0.921	*p* = 0.663	*p* = 0.262	
First	37.4	88.2	1.9	209
Second	36.3	88.3	3.3	244
Third	38.1	85.8	4.6	241

Table 2 shows that obese individuals were more likely to have two or more teeth with gingival bleeding. However, after adjusting for confounding factors, the magnitude of the association was reduced and was not statistically significant [OR (obese × 2 or more teeth) 1.72 (95% CI: 0.95, 3.11)]. After adjusting for potential mediators oral hygiene plus C-reactive protein (data not shown), the magnitude of association with obesity decreased by 47% [OR (obese × 2 or more teeth) 1.38 (95% CI: 0.68, 2.80)]. Concerning waist circumference, the chance of having two or more teeth with gingival bleeding was higher in individuals with waist circumference at level 1 and this association remained even after adjustment for confounding factors [OR (level 1 × 2 or more teeth), 2.03 (95% CI: 1.20, 3.45)], whereas among subjects with waist circumference at level 2 the OR was of 1.36. As for BMI, C-reactive protein was the mediating factor that most accounted for the reduction in the magnitude of the association between waist circumference and gingival bleeding, but the proportion of the effect that was mediated by low-grade inflammation was lower than that observed for overall obesity. The number of episodes of obesity was not associated with risk for bleeding gums.

**Table 2:** Crude and adjusted odds ratios for number of teeth with gingivitis for 24 years according to obesity measurements. Pelotas, RS (2006)

	For number of teeth with gingivitis									
	
	Crude		Adjusted for confounders^3^		Adjusted for oral hygiene reported		Adjusted for C-reactive protein level		Adjusted for percentage of calories from carbohydrates	
					
	1 tooth	2 or more	1 tooth	2 or more	1 tooth	2 or more	1 tooth	2 or more	1 tooth	2 or more
	OR (CI)	OR (CI)	OR (CI)	OR (CI)	OR (CI)	OR (CI)	OR (CI)	OR (CI)	OR (CI)	OR (CI)
Body mass index at 23 years of age	*p* = 0.998^1^	*p* = 0.030^1^	*p* = 0.907^1^	*p* = 0.076^1^	*p* = 0.927^1^	*p* = 0.130^1^	*p* = 0.928^1^	*p* = 0.294^1^	*p* = 0.919^1^	*p* = 0.070^1^
<25 kg/m^2^	Reference	Reference	Reference	Reference	Reference	Reference	Reference	Reference	Reference	Reference
≥25 and ≤ 29.9 kg/m^2^	1.41 (0.81; 2.44)	1.18 (0.77; 1.81)	1.49 (0.86; 2.60)	1.15 (0.74; 1.78)	1.48 (0.85; 2.59)	1.11 (0.71; 1.73)	1.43 (0.78; 2.54)	1.07 (0.67; 1.72)	1.49 (0.85; 2.59)	1.15 (0.67; 1.72)
≥30 kg/m^2^	0.55 (0.16; 1.85)	1.93 (1.08; 3.43)	0.55 (0.16; 1.88)	1.72 (0.95; 3.11)	0.55 (0.16; 1.88)	1.60 (0.88; 2.90)	0.41 (0.09; 1.89)	1.49 (0.74; 3.00)	0.55 (0.16; 1.87)	1.74 (0.96; 3.15)
Waist circumference at 23 years of age	*p* = 0.449^1^	*p* = 0.014^1^	*p* = 0.405^1^	*p* = 0.060^1^	*p* = 0.398^1^	*p* = 0.096^1^	*p* = 0.321^1^	*p* = 0.279^1^	*p* = 0.418^1^	*p* = 0.060^1^
Normal	Reference	Reference	Reference	Reference	Reference	Reference	Reference	Reference	Reference	Reference
Level 1	0.41 (0.12; 1.37)	2.20 (1.31; 3.72)	0.40 (0.12; 1.34)	2.03 (1.20; 3.45)	0.40 (0.12; 1.33)	1.99 (1.16; 3.39)	0.37 (0.11; 1.28)	1.82 (1.03; 3.23)	0.40 (0.12; 1.34)	2.06 (1.21; 3.50)
Level 2	0.92 (0.37; 2.27)	1.56 (0.86; 2.83)	0.89 (0.36; 2.21)	1.36 (0.74; 2.49)	0.88 (0.36; 2.21)	1.28 (0.69; 2.37)	0.77 (0.26; 2.23)	1.07 (0.50; 2.31)	0.90 (0.36; 2.24)	1.36 (0.74; 2.50)
Episodes of obesity	0.109^1^	0.428^1^	0.106^1^	0.566^1^	0.103^1^	0.628^1^	0.062^1^	0.850^1^	0.107^1^	0.567^1^
None	Reference	Reference	Reference	Reference	Reference	Reference	Reference	Reference	Reference	Reference
One episode	0.40 (0.09; 1.70)	1.35 (0.68; 2.65)	0.36 (0.08; 1.56)	1.26 (0.68; 2.65)	0.36 (0.08; 1.56)	1.24 (0.61; 2.52)	0.40 (0.09; 1.79)	1.20 (0.54; 2.68)	0.36 (0.08; 1.55)	1.26 (0.62; 2.54)
Two or more episodes	0.41 (0.10; 1.77)	1.20 (0.59; 2.45)	0.43 (0.10; 1.86)	1.14 (0.59; 2.45)	0.42 (0.10; 1.84)	1.11 (0.53; 2.31)	0.21 (0.03; 1.59)	0.84 (0.36; 1.95)	0.43 (0.10; 1.88)	1.14 (0.55; 2.36)

OR, odds ratio; IC, confidence interval 95%.

1 Wald test for linear trend;

^2^Wald test heterogeneity;

3 Body mass index adjusted for family income, schooling and skin colour; waist circumference adjusted for schooling and skin colour; follow-ups with obesity adjusted for family income, schooling and skin colour.

Dental calculus was also associated with obesity (Table 3), and this association was not mediated by hygiene, inflammation and diet. The number of episodes of obesity from 15 to 23 years of age had a cumulative effect on the presence of calculus, those with two or more episodes had a 13% increased risk of presenting calculus [PR 1.13 (95% CI: 1.06, 1.20)], after adjusting for confounding factors.

**Table 3:** Crude and adjusted prevalence ratios for presence of calculus at 24 years according to obesity measurements. Pelotas, RS (2006)

	Crude	Adjusted for confounders^3^	Adjusted for oral hygiene reported	Adjusted for C-reactive protein level	Adjusted for percentage of calories from carbohydrates
					
	PR (CI)	PR (CI)	PR (CI)	PR (CI)	PR (CI)
Body mass index at 23 years of age	*p* = 0.009^1^	*p* = 0.014^1^	*p* = 0.012^1^	*p* = 0.026^1^	*p* = 0.014^1^
<25 kg/m^2^	Reference	Reference	Reference	Reference	Reference
≥25 and ≤ 29.9 kg/m^2^	1.04 (0.98; 1.11)	1.04 (0.98; 1.11)	1.05 (0.99; 1.12)	1.05 (0.98; 1.12)	1.04 (0.98; 1.11)
≥30 kg/m^2^	1.11 (1.03; 1.19)	1.10 (1.02; 1.18)	1.09 (1.02; 1.17)	1.09 (1.01; 1.18)	1.10 (1.02; 1.18)
Waist circumference at 23 years of age	*p* = 0.031^1^	*p* = 0.063^1^	*p* = 0.055^1^	*p* = 0.157^1^	*p* = 0.059^1^
Normal	Reference	Reference	Reference	Reference	Reference
Level 1	1.01 (0.92; 1.11)	1.02 (0.93; 1.12)	1.01 (0.92; 1.10)	1.02 (0.92; 1.12)	1.01 (0.91; 1.10)
Level 2	1.11 (1.02; 1.17)	1.08 (1.01; 1.15)	1.08 (1.01; 1.16)	1.07 (0.99; 1.17)	1.09 (1.01; 1.16)
Episodes of obesity	<0.001^2^	<0.001^2^	<0.001^2^	<0.001^2^	<0.001^2^
None	Reference	Reference	Reference	Reference	Reference
One episode	1.05 (0.94; 1.16)	1.03 (0.93; 1.15)	1.04 (0.93; 1.15)	1.01 (0.88; 1.15)	1.04 (0.94; 1.15)
Two or more episodes	1.13 (1.06; 1.20)	1.13 (1.06; 1.20)	1.12 (1.06; 1.19)	1.13 (1.09; 1.18)	1.13 (1.06; 1.20)

PR, prevalence ratio; CI, confidence interval of 95%.

1 Wald test for linear trend.

2 Wald test heterogeneity.

3 Body mass index: adjusted for asset index and schooling, waist circumference: adjusted for asset index and schooling; obesity episodes: adjusted for asset index and schooling.

Waist circumference was also associated with the presence of dental calculus and individuals at level 2 had a higher risk [PR 1.08 (95% CI: 1.01, 1.15)]. Adjustment for mediating variables did not alter the magnitude of the association.

Presence of periodontal pockets was not associated with obesity or waist circumference (Table 4) and could be seen by small prevalence ratios and/or large confidence interval. Tests for interactions between BMI and sex, smoking and BMI and waist circumference and smoking were not significant (results not shown).

**Table 4:** Crude and adjusted prevalence ratios for presence of periodontal pockets at 24 years according to obesity measurements. Pelotas, RS

	Crude	Adjusted for confounders^3^	Adjusted for oral hygiene reported	Adjusted for C-reactive protein level	Adjusted for percentage of calories from carbohydrates
					
	PR (CI)	PR (CI)	PR (CI)	PR (CI)	PR (CI)
Body mass index at 23 years of age	*p* = 0.998^1^	*p* = 0.997^1^	*p* = 0.908^1^	*p* = 0.951^1^	*p* = 0.950^1^
<25 kg/m^2^	Reference	Reference	Reference	Reference	Reference
≥25 and ≤ 29.9 kg/m^2^	0.97 (0.36; 2.62)	1.01 (0.38; 2.72)	1.03 (0.39; 2.73)	0.85 (0.30; 2.41)	1.02 (0.38; 2.74)
≥30 kg/m^2^	1.03 (0.24; 4.36)	0.99 (0.25; 4.00)	1.08 (0.28; 4.22)	1.23 (0.26; 5.85)	1.03 (0.26; 4.15)
Waist circumference at 23 years of age	*p* = 0.533^1^	*p* = 0.619^1^	*p* = 0.490^1^	*p* = 0.817^1^	*p* = 0.608^1^
Normal	Reference	Reference	Reference	Reference	Reference
Level 1	1.85 (0.64; 5.35)	1.91 (0.66; 5.53)	2.25 (0.77; 6.65)	2.01 (0.63; 6.44)	1.85 (0.65; 5.27)
Level 2	1.11 (0.26; 4.70)	0.99 (0.25; 4.00)	1.06 (0.28; 4.06)	0.68 (0.09; 5.04)	1.02 (0.26; 4.06)
Episodes of obesity	0.845^2^	*p* = 0.939^2^	*p* = 0.908^2^	*p* = 0.981^2^	*p* = 0.929^2^
None	Reference	Reference	Reference	Reference	Reference
One episode	1.44 (0.35; 5.97)	1.24 (0.32; 4.85)	1.31 (0.34; 5.11)	0.82 (0.11; 6.27)	1.28 (0.33; 4.92)
Two or more episodes	0.78 (0.11; 5.65)	0.87 (0.12; 6.04)	0.85 (0.13; 5.37)	0.93 (0.13; 6.52)	0.89 (0.13; 6.07)

PR, prevalence ratio; CI, confidence interval of 95%.

1 Wald test for linear trend.

2 Wald test for heterogeneity.

3 Body mass index: adjusted for family income and asset index tertiles, waist circumference: adjusted for family income and asset index tertiles; episodes obesity: adjusted for family income and asset index in tertiles.

## Discussion

The present findings suggest that gingivitis in two or more teeth is related to obesity, and this association is partly mediated by oral hygiene and systemic low-grade inflammation. In relation to dental calculus, waist circumference and obesity showed prevalence ratios of similar magnitude. On the other hand, the presence of periodontal pockets was neither related to obesity nor to waist circumference. A cumulative effect of obesity was only observed for dental calculus.

The subjects in this study are a representative sample of young adults participating in a birth cohort, the percentage of losses to follow up was small. Because the probability of being followed in the Oral Health Survey – 2006 (mean age 24 years) was not associated with obesity at 23 years, we believe that this study is not susceptible to selection bias. The sequential measurements of obesity in this study allowed for analysis of the accumulation of risk of obesity in relation to the outcomes studied. Anthropometric data were collected by trained examiners and standardized at all visits, reducing the possibility of measurement error.

By using only the measurement of periodontal pocket to assess the presence of periodontitis its prevalence may have been underestimated due to possible clinical attachment loss without the presence of the pocket. However, the measure of outcome is similar to that often used in other studies (Ekuni et al. 2008, Cinar & Murtomaa 2011, Morita et al. 2011). The low prevalence reduces the power of the study, limiting the capacity to find a statistically significant association. Another limitation of this study was the marked asymmetry of continuous data on gingivitis and periodontal pocket that led to the generation of categories for statistical analysis. An additional limitation relates to the variable consumption of carbohydrates. This variable assessed the total intake of carbohydrates and did not discriminate against obesogenic and dental plaque formation potential which may have influenced the estimate of the mediating effect. The lack of association between periodontitis and obesity cannot be attributed to its low prevalence, as we did not observe a linear trend according to nutritional status. It is suggested that in young adults, the presence of obesity may not be related to the occurrence of periodontal pockets corroborating previous studies where obesity in young adults was not associated with periodontal disease in later ages (Linden et al. 2007).

The lack of a statistically significant association between gum disease and obesity may be due to the fact that the prevalence of gingivitis was close to 50% (37.7%). Resulting in higher variance for the estimates and consequently in larger confidence interval and reduced statistical power, even though the magnitude of effect was greater than that observed to the other outcomes. The risk of having one tooth affected was not associated with BMI, whereas the risk of more extensive gingival bleeding (two or more teeth affected) was positively associated with BMI. C-reactive protein was one of the mediators of the association between gingivitis and obesity suggesting that the presence of a low-grade systemic inflammation due to obesity may trigger increased local inflammatory response to external stimuli such as dental plaque. These findings are consistent with the results observed by Wu et al. (2000) who found that C-reactive protein levels are positively associated with the extent of gingivitis. The adjustments for both mediating factors, oral hygiene and C-reactive protein, also suggest an independence of these two mechanisms, where each one has a distinct share in the process of gingival inflammatory response.

Similar to previously reported findings, dental calculus was associated with BMI and waist circumference (Wood et al. 2003). The presence of supragingival calculus is a risk factor for periodontitis in young (Susin et al. 2011). Given the characteristics of dental calculus to promote the accumulation of plaque and its maintenance, the presence of dental calculus more often in young individuals who are obese may be a sign of severity of periodontal disease (periodontitis; Van Der Velden 2006). Further studies are needed to verify whether those who are exposed to risk factors for a longer period of time will eventually have periodontitis with greater frequency and severity.

Although no association was found between measures of obesity and periodontitis, this study identified an association between the number of episodes of obesity and risk for dental calculus and also between waist circumference and the number of teeth with gingivitis. It should be kept in mind that gingivitis is an indicator of poor hygiene condition. The dental calculus may have an important role in the development and aggravation of periodontitis by predisposing plaque formation and maintenance along the gingival margin. The combination of these two conditions in obese subjects suggests that these individuals may be at greater risk of chronic periodontitis. Therefore, obese subjects should not only be targeted of health promotion activities for co-morbidities such as diabetes but also to evaluate oral health conditions, especially periodontal disease.

This study was able to identify the mediating effect of a marker of systemic inflammation or oral hygiene in the association between obesity and gingival bleeding. The observation that inflammation is a mediator in the association between obesity and gum disease confirms the hypothesis raised by Pischon et al. (2007). According to our knowledge, this is the first study to report such mediation.

**Clinical Relevance**

*Scientific rationale for the study*: Association between obesity and periodontal disease has been reported mainly in cross-sectional studies and the mechanisms of this association have been scarcely studied. Longitudinal studies that evaluate potential mediators of this association are needed.

*Principal findings*: Body mass index and waist circumference were associated with gingivitis and dental calculus, but not with periodontal pocket. Part of the association is mediated by systemic inflammation in young adults.

*Practical Implications*: Young obese have a higher risk of developing gingivitis and dental calculus, risk factors for periodontitis. Further follow-up periods are needed to assess the incidence of periodontitis.
